# *In vivo* treatment with epigenetic modulating agents induces transcriptional alterations associated with prognosis and immunomodulation in multiple myeloma

**DOI:** 10.18632/oncotarget.3207

**Published:** 2014-12-26

**Authors:** Ken Maes, Eva De Smedt, Alboukadel Kassambara, Dirk Hose, Anja Seckinger, Els Van Valckenborgh, Eline Menu, Bernard Klein, Karin Vanderkerken, Jérôme Moreaux, Elke De Bruyne

**Affiliations:** ^1^ Department of Hematology and Immunology, Myeloma Center Brussels, Vrije Universiteit Brussel, Brussels; ^2^ Department of Biological Haematology, CHU Montpellier, Montpellier, France; ^3^ Institute of Human Genetics, CNRS-UPR1142, Montpellier F-34396, France; ^4^ Medizinische Klinik, Universitätsklinikum Heidelberg, Heidelberg, Germany; ^5^ University of Montpellier 1, UFR de Médecine, Montpellier, France

**Keywords:** Epigenetics, Multiple Myeloma, HDACi, DNMTi, murine model

## Abstract

Histone deacetylase inhibitors (HDACi) and DNA methyltransferase inhibitors (DNMTi) are in early clinical development for multiple myeloma (MM) therapy. Despite all encouraging pre-clinical data, clinical activity of HDACi and DNMTi is mostly lacking. To optimize the trials, characterization of the *in vivo* response towards HDACi and DNMTi will be crucial. Therefore, we investigated the transcriptional response after *in vivo* treatment with the HDACi quisinostat or DNMTi decitabine using the murine 5T33MM model.

We identified 504 and 154 genes deregulated by quisinostat and decitabine, respectively. Of interest, MM patients' gene expression levels of 62 quisinostat- and 25 decitabine-deregulated genes were predictive for overall survival of patients. This prognostic information was implemented in a DNA methylation and histone acetylation score. A high score was related to a high proliferative and immature phenotype of MM cells. Furthermore, highly scored MM patients had an adverse overall survival. Interestingly, bio-informatic prediction tools revealed an association of quisinostat-deregulated genes with lymphocyte activation, proliferation, immune-effector mechanisms and T-helper-1 development.

Overall, treatment of 5T33MM mice with epigenetic modulating agents led to the translation of gene signatures to predict overall survival of MM patients. HDACi mainly deregulated tumoral immunomodulatory pathways, supporting the rationale to combine HDACi with immunomodulatory therapies.

## INTRODUCTION

Multiple myeloma (MM) is a hematological plasma cell malignancy. Malignant plasma cells mainly reside in the bone marrow (BM) where numerous interactions between MM cells and the BM compartments confer survival and growth of MM cells and induce angiogenesis, bone destruction, drug resistance and immune escape [1]. MM is characterized by a genetic heterogeneity that is translated to a large range of patients' survival times [2]. Prognosis of MM patients can be assessed by the International Staging System reflecting disease activity and inflammatory status. The molecular characteristics of MM cells are determined by chromosomal alterations and alterations in gene expression [3-11]. Treatments consist of combinations of various drug classes such as glucocorticoids, proteasome inhibitors, alkylators and immunomodulatory drugs (IMiDs) with or without high dose melphalan (HDM) and autologous stem cell transplantation (ASCT) [12]. Although the combination of these drugs have significantly improved patient survival [13-15], a majority of patients still relapses, emphasizing the need to find alternative treatment options.

Epigenetic aberrations have been reported to contribute to MM pathogenesis, together with genetic abnormalities [16-18]. DNA methylation and posttranslational histone modification are two major epigenetic modifications. DNA methylation is considered a prognostic marker in human MM [19-23] and differences can be detected between MGUS and MM in terms of gene-specific methylation and global hypomethylation of non-CpG islands (repetitive elements or intergenic regions) [22, 24]. Moreover, cytogenetic MM subgroups are defined by differences in DNA methylation patterns [22, 25]. Among posttranslational histone modifications, methylation and acetylation are extensively studied in relation to cancer [26]. The t(4;14) translocation in MM cells, present in approximately 15% of the patients, leads to overexpression of *WHSC1* encoding for the histone methyltransferase MMSET [27]. In addition, mutations in the histone methyltransferases *WHSC1L1*, *MLL1-3* and in the histone demethylase *UTX* are identified in MM patients [28, 29].

Epigenetic modulating agents such as histone deacetylase inhibitors (HDACi) and DNA methyltransferase inhibitors (DNMTi) interfere with epigenetic aberrations in cancer [30]. HDACi used alone or in combination with conventional anti-MM agents have potent pre-clinical anti-MM effects [31-33]. The same holds true for the DNMTi azacytidine or decitabine [34, 35]. Furthermore, using HDACi and DNMTi, we recently identified gene expression-based risk scores, which are predictive for the sensitivity of MM cells towards DNMTi and HDACi as well as for the overall survival of MM patients. This pre-clinical work provides the rationale for clinical trials evaluating the anti-MM activity of HDACi. While single-agents HDACi appeared to mediate little to no clinical activity [36-38], combinatory treatment of the pan-HDACi vorinostat or panobinostat in combination with respectively the proteasome inhibitor bortezomib or bortezomib plus dexamethasone did prolong progression free survival with respectively 0.8 months and 3.9 months [39, 40]. However, the clinical relevance in terms of overall survival is not yet clear and a high occurrence of side effects was observed [39, 40]. In addition, a phase II trial of the combination of panobinostat with melphalan, thalidomide and prednisone was also associated with a high occurrence of side effects [41]. As for the DNMTi, the therapeutic potential in MM is yet to be evaluated in clinical trials.

These above mentioned clinical trials raise questions about the efficacy of epigenetic modulating agents in MM patients. Although the pre-clinical studies on epigenetic modulating agents demonstrated pleiotropic mechanisms of action explaining their anti-MM activity [34, 42-44], it is widely known that the drug response of MM cells is influenced by interactions with the BM microenvironment and immune system [45-47]. Thus, a better understanding of the *in vivo* mechanisms of epigenetic modulating agents will be crucial as it can provide new possibilities for combinatory therapies, identify more specific targets, reduce side effects and identify the patients whom might benefit from treatment with epigenetic modulating agents [48]. We have previously demonstrated potent *in vivo* anti-MM activity for decitabine and the HDACi quisinostat using the immune competent, syngeneic 5TMM models [31, 32, 34]. These models are suitable for studies on MM biology and pre-clinical drug testing because they take into account the BM microenvironment and immune system [32, 49-52]. Here, we investigated the *in vivo* transcriptional response of MM cells towards decitabine and quisinostat in the 5T33MM model to validate our previous work on the prognostic relevance and to identify new *in vivo* relevant targets.

## RESULTS

### *In vivo* treatment with epigenetic modulating agents induced transcriptional changes linked with survival of MM patients

The syngeneic immunocompetent 5T33MM model was used to study the *in vivo* transcriptional response towards the DNMTi decitabine and the HDACi quisinostat. Quisinostat is a hydroxamate-based pan-HDAC inhibitor with similar HDAC selectivity as panobinostat [53]. In a preliminary series of experiments, the sub-lethal concentrations showing minimal effects on BM plasmacytosis ensuring the yield of good quality RNA were determined (data not shown). Next, mice with established disease were treated with these sub-lethal concentrations of decitabine or quisinostat for 5 days (Supplementary Figure S1A). Total BM plasmacytosis was above 80% in the vehicle and decitabine-treated mice, while quisinostat decreased tumor load to approximately 60% (Supplementary Figure S1B). Overall, the short-term treatment had only minimal anti-MM effects thus warranting good sample quality.

**Figure 1:**
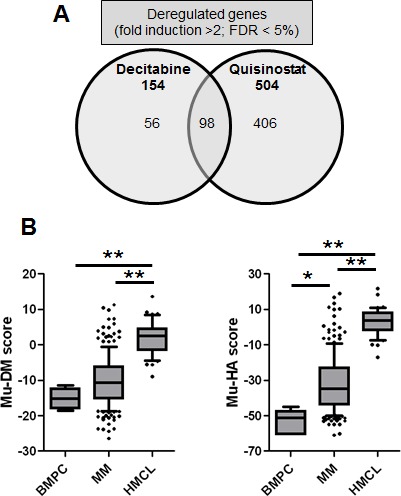
Overview of the *in vivo* transcriptional response towards decitabine or quisinostat and the gene expression-based risk scores A: Venn-diagram of deregulated genes after *in vivo* treatment of the 5T33MM model with decitabine or quisinostat. Microarray data were normalized using MAS5 and analyzed using SAM for the identification of differentially expressed probe sets (ratio ≥ 2, Benjamini-Hochberg p value < 0.05). B: The Mu-DM and Mu-HA score during MM progression. The prognostic value of decitabine- or quisinostat-deregulated genes was calculated using Maxstat. Decitabine and quisinostat led to deregulation of respectively 25 and 61 prognostic genes which were used to develop a gene expression-based risk score as explained in Material and Methods. The boxes represent median and 10-90 percentiles of the score values during MM progression. * = p<0.05, ** p<0.001. BMPC = healthy donor bone marrow plasma cell, MM = multiple myeloma, HMCL = human myeloma cell lines.

**Figure 2:**
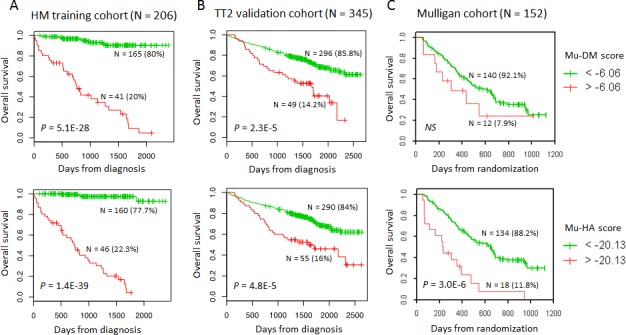
The prognostic value of the Mu-DM and Mu-HA score in terms of overall survival A: Using Maxstat analysis, the prognostic value of Mu-DM (top) and Mu-HA (bottom) score was calculated by the optimal separation of patients of the HM-cohort (n=206) in a low and high risk group based on a cut-off value (−6.06 for Mu-DM score; −20.13 for Mu-HA score). B: The prognostic value of the Mu-DM (top) and Mu-HA (bottom) score were tested in the independent TT2-cohort (n=345) using the same cut-off value as obtained in the HM-cohort. C: The prognostic value of the Mu-DM (top) and Mu-HA (bottom) score were tested in the independent Mulligan-cohort using the same cut-off value as obtained in the HM-cohort.

Using Significance Analysis of Microarray (SAM), decitabine treatment resulted in a significant upregulation of 172 probe sets and downregulation of 8 ones (false discovery rate (FDR) ≤ 5%; ratio ≥ 2), corresponding to 154 unique genes. Quisinostat treatment induced a significant upregulation of 569 probe sets and downregulation of 5 probe sets (FDR ≤ 5%; ratio ≥ 2), corresponding to 504 unique genes. Ninety-eight genes were commonly deregulated by decitabine and quisinostat (Figure 1, Supplementary Tables S1-S2). The prognostic value of the human orthologs for the overall survival of patients with MM of murine deregulated genes was investigated using patients data from the University Clinics of Heidelberg and Montpellier (HM)-cohort as a training cohort and the University of Arkansas for Medical Sciences-Total Therapy 2 (TT2-cohort) as validation cohort [54-57]. Using the HM-cohort, five decitabine-deregulated probe sets had a prognostic value for a better overall survival and 20 for an adverse one (Table 1). Thirty-one quisinostat-deregulated genes had a favourable prognostic value and 30 an adverse one (Table 1). The prognostic information of the 25 decitabine- or 61 quisinostat-deregulated genes was implemented into respectively a murine DNA methylation (Mu-DM) score or a murine histone acetylation (Mu-HA) score, as described in Material and Methods. Subsequently, we analyzed the scores during MM progression [58]. MM cells had a higher Mu-HA score (p<0.05) compared to purified healthy plasma cells (BMPCs). HMCLs had significant higher scores (p<0.0001) compared to MM cells and BMPCs (Figure 1). Both scores could predict patients' overall survival. In the training cohort, a maximum difference in overall survival (OS) was identified with a Mu-DM score of −6.06 (Figure 2A). Patients with Mu-DM score ≤ −6.06 had a significant lower risk (80% of the patients who did not reach median OS) compared to patients with a Mu-DM score > −6.06 (20% of the patients with a median OS of 25 months). For the Mu-HA score, a maximal difference in OS was observed with −20.13 as cut-off, splitting patients in a low-risk group (Mu-HA score ≤ −20.13; 77.7% of the patients who did not reach median OS) and a high-risk group (Mu-HA score > −20.13; 22.3% of the patients with a median survival of 24 months) (Figure 2A). This prognostic value of the score was validated in an independent validation cohort (TT2-cohort) as shown in Figure 2B. We then evaluated whether the scores could have a prognostic value in relapsed patients. Using the Mulligan-cohort, including patients treated with bortezomib after relapse, only the Mu-HA score kept prognostic value (Figure 2C). Next, we evaluated whether the scores could be used to predict response to therapy. Using the HM-cohort, we could not find a significant difference in the distributions of low scored and high scored patients between different response groups following high-dose therapy and autologous stem cell transplantation (data not shown). For the Mulligan-cohort we only tested the Mu-HA score. The distributions of low scored and high scored patients were significantly different between non-responders (no change + progressive disease) compared to responders (complete response + partial response + minimal response) (Supplementary Table S3). The prediction had a high specificity (97.3%), but a low sensitivity (16.1%).

**Table 1:** Prognostic value of the deregulated genes as determined by Maxstat analysis The human orthologs of the *in vivo* deregulated murine genes after treatment with quisinostat or decitabine were used to determine the prognostic value in the HM training cohort

Decitabine			
Probeset	Name	Benjamini-Hochberg adjusted p-value (FDR)	Hazard ratio
Good prognostic genes (5)			
203708_at	PDE4B	0.016	0.184
225629_s_at	ZBTB4	0.051	0.346
217492_s_at	PTENP1	0.049	0.358
202917_s_at	S100A8	0.034	0.363
215210_s_at	DLST	0.049	0.405
Bad prognostic genes (20)			
219684_at	RTP4	0.046	5.158
224701_at	PARP14	0.002	4.106
217503_at	STK17B	0.010	3.796
222848_at	CENPK	0.007	3.743
223271_s_at	CTDSPL2	0.007	3.681
228351_at	HEATR1	0.046	3.633
204709_s_at	KIF23	0.012	3.550
213647_at	DNA2	0.009	3.537
219211_at	USP18	0.020	3.140
212416_at	SCAMP1	0.048	2.981
212577_at	SMCHD1	0.024	2.782
208901_s_at	TOP1	0.026	2.753
224227_s_at	BDP1	0.041	2.711
242625_at	RSAD2	0.049	2.701
213742_at	SRSF11	0.036	2.611
218585_s_at	DTL	0.036	2.582
228006_at	PTEN	0.045	2.580
225647_s_at	CTSC	0.045	2.554
243213_at	STAT3	0.046	2.538
226942_at	PHF20L1	0.047	2.382
**Quisinostat**			
Probeset	Name	Benjamini-Hochberg adjusted p-value (FDR)	Hazard ratio
Good prognostic genes (31)			
223806_s_at	NAPSA	0.048	0.153
222717_at	SDPR	0.045	0.184
203708_at	PDE4B	0.029	0.184
210889_s_at	FCGR2B	0.008	0.237
224826_at	GPCPD1	0.009	0.240
202878_s_at	CD93	0.012	0.261
201445_at	CNN3	0.007	0.270
200934_at	DEK	0.020	0.281
212298_at	NRP1	0.013	0.302
201243_s_at	ATP1B1	0.044	0.314
224586_x_at	SUB1	0.046	0.322
210664_s_at	TFPI	0.018	0.326
235593_at	ZEB2	0.050	0.342
207794_at	CCR2	0.046	0.347
225175_s_at	SLC44A2	0.039	0.349
224964_s_at	GNG2	0.045	0.353
203799_at	CD302 /// LY75-CD302	0.045	0.355
224983_at	SCARB2	0.043	0.358
212268_at	SERPINB1	0.044	0.359
222391_at	TMEM30A	0.036	0.360
209916_at	DHTKD1	0.039	0.363
202917_s_at	S100A8	0.048	0.363
209829_at	FAM65B	0.039	0.367
200965_s_at	ABLIM1	0.044	0.374
226841_at	MPEG1	0.050	0.375
219062_s_at	ZCCHC2	0.045	0.382
224442_at	PHF6	0.044	0.388
202990_at	PYGL	0.049	0.390
205790_at	SKAP1	0.045	0.402
215210_s_at	DLST	0.049	0.405
226925_at	ACPL2	0.049	0.409
Bad prognostic genes (30)			
204040_at	RNF144A	0.043	5.358
217503_at	STK17B	0.017	3.796
223271_s_at	CTDSPL2	0.010	3.681
203091_at	FUBP1	0.049	3.336
241879_at	LPP	0.044	3.195
203449_s_at	TERF1	0.047	3.195
215111_s_at	TSC22D1	0.040	3.133
212151_at	PBX1	0.043	3.078
203636_at	MID1	0.020	3.046
201912_s_at	GSPT1	0.038	2.985
212416_at	SCAMP1	0.049	2.981
209798_at	NPAT	0.044	2.944
233011_at	ANXA1	0.043	2.944
222133_s_at	PHF20L1	0.038	2.860
204809_at	CLPX	0.035	2.860
223319_at	GPHN	0.037	2.789
212577_at	SMCHD1	0.036	2.782
208901_s_at	TOP1	0.038	2.753
224227_s_at	BDP1	0.045	2.711
1557737_s_at	NKTR	0.050	2.651
208831_x_at	SUPT6H	0.050	2.640
224848_at	CDK6	0.047	2.625
213742_at	SRSF11	0.048	2.611
218585_s_at	DTL	0.043	2.582
214953_s_at	APP	0.046	2.581
228006_at	PTEN	0.046	2.580
225647_s_at	CTSC	0.049	2.554
220994_s_at	STXBP6	0.045	2.546
225097_at	HIPK2	0.043	2.539
202446_s_at	PLSCR1	0.049	2.447

Next, the prognostic value for overall survival of Mu-DM and Mu-HA scores was compared with conventional prognostic factors including beta-2-microglobulin (B2m), International Staging (ISS), t(4;14) and del17p. We also included other gene expression-based risk scores such as High Risk Score (HRS corresponding to UAMS-70-gene model) [8], Intergroupe Francophone du Myélome (IFM) score [4], Risk Score (RS) [7], DNA methylation (DM) score [35], histone acetylation (HA) score [59] and gene expression-based proliferation index (GPI) [5]. Univariate Cox analysis showed that all these factors have prognostic value (Supplementary table S4) [5, 35, 59]. When analyzed 2 by 2, Mu-DM remained significant with del17p and Mu-HA score in the HM training cohort. The Mu-HA score remained significant with t(4;14) and Mu-DM in bivariate analyses. Multivariate Cox analysis with all parameters tested together showed that Mu-DM, Mu-HA and t(4;14) remained independent prognostic factors in the HM training cohort. In the TT2 validation cohort, Cox bivariate analysis showed significance of Mu-DM and Mu-HA score together with all factors including the previously reported DM and HA scores but not with HRS and IFM scores (Supplementary table S4). HRS and t(4;14) remained independent prognostic factors when all parameters were tested together in the TT2 validation cohort. When tested with the previously reported DM and HA score, the Mu-DM and Mu-HA score remained independent in the HM validation cohort but not in the TT2 training cohort in the multivariate analysis (Supplementary Table S4).

**Figure 3:**
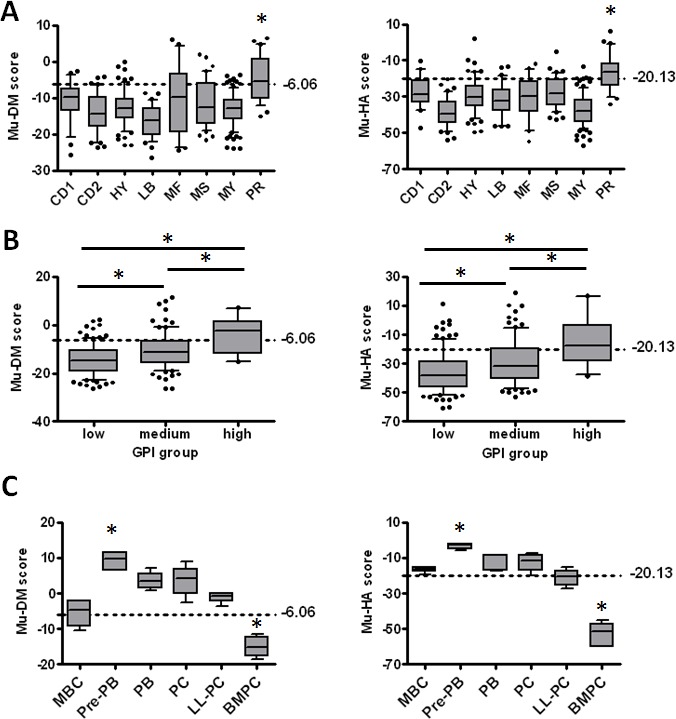
The score values in the different MM molecular subgroups, gene expression-based proliferation index (GPI) subgroups and during B-cell development A: The Mu-DM (left) and Mu-HA (right) score values were calculated based on the gene expression data of the 8 MM molecular subgroups of the TT2-cohort. PR = proliferation, LB = low bone disease, MS = MMSET, HY = hyperdiploid, CD1 = cyclin D1, CD2 = cyclin D2, MF = MAF; MY = myeloid. * indicates higher score value compared to all other groups with p<0.05. B: The Mu-DM (left) and Mu-HA (right) score values were calculated in the different gene expression-based proliferation index groups (GPI). * = p<0.05. C: The score values in the different stages of *in vitro* generated plasma cell differentiation. MBC = memory B-cell, PPB = pre-plasmablast, PB = plasmablast, PC = early plasma cell, LL-PC = long-lived plasma cell, BMPC = healthy donor bone marrow plasma cell. * indicates p<0.05 compared to all other groups. The boxes represent median and 10-90 percentiles of the score values. Dots are outliers and the dotted lines represent the cut-off value for each score.

To investigate the characteristics of the stratified patients, the distribution of the Mu-DM and the Mu-HA score among the 8 molecular subgroups of MM using the TT2-cohort was analyzed [60]. Both scores were significantly higher (p<0.05) in the proliferation subgroup (PR) compared to all other subgroups (Figure 3A). The PR subgroup was the only group with a median score above the cut-point and has been associated with a poor prognosis [60]. In addition, a significant correlation between GPI groups from HM-cohort and Mu-DM or Mu-HA score was identified (r = 0.36; p<.01; n=206 and r = 0.32; p< .01; n=206 respectively) in MM cells of patients. This is further confirmed by the significant gradual increase in the Mu-DM and Mu-HA scores from GPI^low^ to GPI^medium^ and GPI^high^ groups (p<0.05) (Figure 3B) [5]. Recently, the presence of a hierarchical plasma cell progenitor organization was described in the BM of MM patients and it was furthermore suggested that an epigenetic-mediated plasticity exists between these plasma cell progenitors [61]. Therefore, we analyzed the scores in the *in vitro* generated transitional stages from memory B-cell to plasma cells [58, 62]. We found that both scores were significantly higher (p<0.05) in pre-plasmablast (Pre-PB) stages and significantly lower (p<0.05) in healthy donor BMPCs compared to all other conditions. The other stages were considered as having an intermediate Mu-DM and Mu-HA score with plasmablasts (PBs), plasma cells (PCs) and long-lived plasma cells (LL-PCs) having a higher Mu-DM score compared to memory B-cells (MBCs) (Figure 3C). In summary, quisinostat and decitabine altered *in vivo* the expression of a gene signature that is associated with prognosis allowing risk stratification of patients. High scores are furthermore linked with a proliferative and immature plasma cell phenotype.

### *In vivo* quisinostat or decitabine treatment altered the expression of genes involved in immune pathways

**Figure 4:**
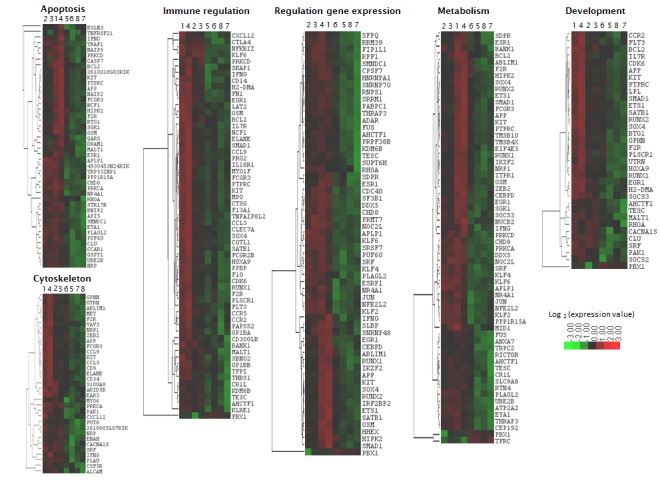
Heatmap of quisinostat-deregulated genes with overlapping functions identified by GO analysis and Reactome Hierarchical clusters were made using Cluster and Treeview. Sample 1-4: quisinostat. Sample 5-8: vehicle.

**Figure 5:**
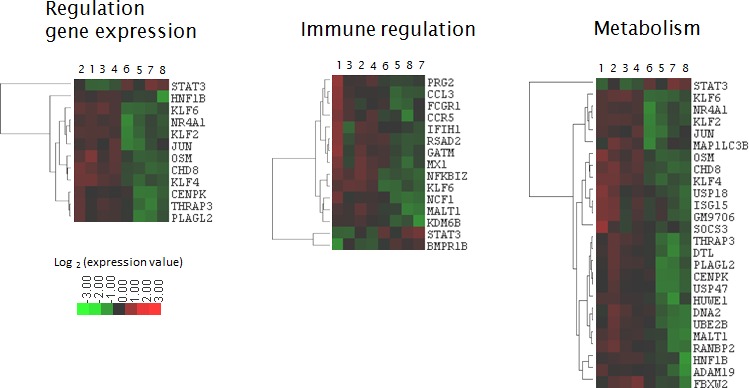
Heatmap of decitabine-deregulated genes with overlapping functions identified by GO analysis and Reactome Hierarchical clusters were made using Cluster and Treeview. Sample 1-4: decitabine. Sample 5-8: vehicle.

Using DAVID gene ontology (GO) software, the enrichment of the *in vivo* deregulated genes with a biological process was investigated [63, 64]. GO analysis showed a significant enrichment of genes involved in immune regulation, metabolism/homeostasis, development and differentiation, cytoskeleton/migration, regulation of gene expression and cell death (Supplementary Table S5). The hierarchical clustering of the co-enriched gene signatures is shown in Figure 4 and 5. Looking to the genes included in the scores in more detail showed that these genes were dispersed across all the different enriched GO sets. STRING protein network analysis confirmed the GO analysis and showed network interactions linked with immune cell regulation, cell death pathways and metabolism (Supplementary Figure S2, S3) [65]. Reactome pathway analysis revealed that after quisinostat treatment, there were several enriched gene sets and these were associated with growth factor signaling including tumor necrosis factor (TNF), TNF-related apoptosis-inducing ligand (TRAIL), interferon-gamma (IFN-γ), interleukin-1 (IL-1), IL-2, IL-12, bone morphogenetic protein (BMP) and transforming growth factor beta (TGF-β). In addition, several signaling pathways showed enrichment of deregulated genes and include p38/MAPK, PI3K/Akt, SMAD, eIF4e, proteoglycan and integrin signaling (Supplementary Table S6).

Of particular interest, GO analysis, Reactome and STRING commonly identified a significant enrichment of genes linked with immune pathways. This immune pathway gene signature was characterized by genes encoding for proteins involved in lymphocyte activation and proliferation, immune-effector processes and T-helper-1 development as depicted by Reactome. Using Pathway-Guide, we found that the gene signature is built up by genes involved in chemokine signaling, cytokine interactions, phagocytosis, T-cell receptor and natural-killer (NK) cell signaling (Supplementary Table S7). The signature includes genes coding for chemokines (C*XCL12, PBPB, CCL9, CCL3*), chemokine receptors (*CCR5, CCR2*), cytokines (*IFNG, OSM*) and cytokine receptors (*IL7R, IL18R1, KIT, FLT3, CSF1R, CSF3R, MET, TNFRSF12*). Genes encoding for proteins involved in lymphocyte activation include co-inhibitory proteins (*CTLA4*, *CTLA2A, CTLA2B*), co-stimulatory proteins (*PTPRC*), antigen-presenting protein (*H2-DMA*), lymphocyte activation marker (*CD69*), monocyte marker (*CD14)*, transcription factors (*RUNX1, PBX1, SMAD1, HOXA9, NFKBIZ, JUN, KLF6*) and NK-cell inhibitory receptors (*KLRD1, KLRA7*). Genes encoding for proteins associated with immune-effector responses include phagocytose receptors (*FCGR3, FCGR2B*), phagocytosis mediators (*PRKCA, PRKCD*), extracellular matrix (ECM) component *FN1* and its receptor *ITGA5* and more downstream regulators (*PAK1, RHOA*, *VAV3*, *IQGAP2, ENAH, APCRB, TMSB4X, DIAP1*) involved in actin polymerization, adherens junctions and stress fibers polymerization (Figure 4, Supplementary Table S5, S6, S7). Thus, *in vivo* treatment with quisinostat induced a broad transcriptional response within the tumor cells with a significant deregulation of genes encoding for proteins involved in immune regulation pathways.

## DISCUSSION

To gain insight into the *in vivo* transcriptional response towards epigenetic modulating agents, we treated the syngeneic immunocompetent murine 5T33MM model with sub-lethal doses of the DNMTi decitabine and the HDACi quisinostat. First, *in vivo* treatment identified several deregulated genes with a prognostic value in the HM-cohort allowing the development of gene expression-based risk scores. In accordance with our previous work in HMCL, both the Mu-DM and Mu-HA score were found to have a prognostic value for OS in two tested independent MM cohorts [35, 59]. In line, both the Mu-DM and Mu-HA score were an independent variable for prognosis of OS together with t(4;14) in the HM-cohort but not in the TT2-cohort after multivariate COX analysis. In addition, the Mu-HA and Mu-DM score were independent of the previous published HA- and DM score in the HM cohort [35, 59]. In addition, we showed that the Mu-HA score is predictive for overall survival of relapsed patients treated with bortezomib and further confirmed that gene expression profiling is useful for the prediction of outcome of newly diagnosed and relapsed patients [66]. The predictive power of the scores for treatment response was limited as the Mu-HA score had a low specificity of prediction for response to bortezomib and we did not identify a significant association between the Mu-DM or Mu-HA score and response to highdose conditioning chemotherapy and autologous stem cell transplantation. This confirms recently published data that gene expression profiling alone is not sufficient for predicting treatment response [67, 68]. Overall, these data show the translation of gene signatures obtained after *in vivo* treatment of mice with epigenetic modulating agents that can be used to predict overall survival of previously untreated and relapsed MM patients. This furthermore validates the use of the 5T33MM model for evaluating the response towards epigenetic modulating agents and provides the framework for testing other epigenetic modulating agents with other or more specific targets.

Subsequently, we studied which factor(s) are responsible for the separation of MM patients. The observation that patients from the “PR” MM subgroup linked with a bad prognosis have higher Mu-DM and/or Mu-HA scores compared to all other subgroups indicates an association of high risk with proliferation and is in line with the results of the *in vitro* obtained DM- and HA-score [35, 59, 69]. The gradual higher score values in the GPI^high^ and GPI^medium^ group compared to the GPI^low^ group furthermore confirm the link between a high score and high proliferation [5]. We also compared transitional populations from memory B-cells to plasma cells for their score values. Pre-plasmablasts and plasmablasts are characterized by a higher proliferation rate compared to memory B-cells, early plasma cells and BMPCs [62, 70]. In addition, HMLCs were developed from primary MM samples which escaped the BM dependency and are highly proliferative [58, 71]. Our observation that pre-plasmablasts and HMCL displayed the highest Mu-DM and Mu-HA scores again indicates a link between a high score and proliferation. Overall, this validates our earlier work with the DM-score and HA-score [35, 59, 69] and suggests an association between the expression levels of epigenetically regulated genes and a high risk linked with a high proliferative and immature plasma cell phenotype.

Recently, the existence of plasma cell progenitors that recapitulate the different maturation stages of plasma cell differentiation was described within the BM of MM patients [62, 70] and was associated with proteasome inhibitor resistance [61, 72]. MM progenitors including B cells and pre-plasmablasts were found to survive treatment with proteasome inhibitors and were significantly enriched in MM patients refractory to bortezomib treatment. These Xbp1s negative pre-plasmablastic cells are characterized by a diminished endoplasmic reticulum stress and thus resistance to proteasome inhibitors since they are not committed to high Ig production [72, 73]. Furthermore, plasmablastic progenitors have been described to overexpress epigenetic regulators, compared to mature plasma cells, suggesting that transitions in plasma cell differentiation stages could be linked to epigenetic plasticity [61]. Thus, HDACi or DNMTi combined treatment could influence the plasticity of plasma cell progenitors and potentially target tumor progenitors that contribute to treatment failure in MM. Moreover, the scores presented here may be useful to predict the presence of such immature plasma cell populations.

In line with previous *in vitro* gene-expression profiling studies, we found associations of *in vivo* HDACi- and DNMTi-deregulated genes with biogenesis, cytoskeletal organization and immunological pathways [20, 35, 59]. In particular, we provide evidence that quisinostat (and to a lesser extend decitabine) induces tumoral transcriptional changes of genes involved in immune response pathways, namely lymphocyte activation and proliferation, immune-effector processes and T-helper-1 development. The immune system is suppressed in MM and studies focusing on reversing the suppressive state to boost immune-mediated anti-tumor responses are ongoing [74]. Emerging evidence indicates that epigenetics plays an important role in various aspects of the immune system including cytokine production, dendritic cell activation, plasticity of CD4+ T cells, regulatory T cell function and plasma cell differentiation [75, 76]. In line, recent pre-clinical work has demonstrated that epigenetic modulating agents have immunomodulatory effects. West *et* al. suggested that HDACi require an intact immune system for long term effects in murine cancer models [43]. The authors demonstrated that the HDACi vorinostat mediated immunogenic anti-tumor effects through IFN-γ production by B-cells [77]. These effects were moreover enhanced by α-galactosylceramide and may imply a role for NKT cells [77, 78]. Vorinostat was also shown to possess immunomodulatory properties in isolated peripheral blood mononuclear cells and in mice after lipopolysaccharide exposure [79]. Interestingly, in AML patients, panobinostat in combination with azacytidine decreased TNFR2+ regulatory T-cell populations associated with IFN-γ and IL-2 induction [80]. Taken together, our data and work by others supports the use of HDACi in combination with immunomodulatory therapies such as IMiDs,transplantation, humoral or cellular/peptide vaccines as recently suggested [77, 81].

So far, a few pre-clinical studies demonstrated combinatory effects of HDACi and IMiDs in MM but lack mechanistic evidence. Vorinostat in combination with the thalidomide derivate IMiD1 showed combinatory effects *in vitro* [82]. In addition, panobinostat enhanced anti-MM effects of lenalidomide and dexamethasone *in vitro* and *in vivo* [83]. Vorinostat or panobinostat have been shown to present synergistic effects in combination with the immune cell stimulating antibodies anti-CD40 and anti-CD137 in immunocompetent models of mammary, renal and colon carcinoma [84]. Very recently, in a murine melanoma model, panobinostat was demonstrated to synergize with an adoptive T-cell transfer leading to systemic immune responses to reduce melanoma tumor burden [81]. Panobinostat influenced T-cell populations and systemic cytokine production independent from the presence of tumor [81]. The above work suggests the potential of combining HDACi with immunotherapy strategies. Nevertheless, future pre-clinical *in vivo* work is mandatory to further address this possibility. Regarding safety issues, it was only recently shown that the combination of vorinostat and lenalidomide plus dexamethasone is well tolerated in MM patients in a phase I clinical trial [85].

In conclusion, *in vivo* treatment of MM cells with epigenetic modulating agents results in a transcriptional response that can be linked to prognosis. This prognostic signature was used to construct a gene expression based risk score allowing risk stratification of newly diagnosed patients. In addition, HDACi treatment (and to a lesser extent DNMTi) resulted in a wide transcriptional response involving overlapping gene signatures mainly mediating immune response. This indicates that transcriptional immune regulation is an important *in vivo* biological response of tumor cells towards HDACi and supports the rationale for the combination of HDACi and immunomodulatory therapies.

## MATERIALS AND METHODS

### Drugs

Decitabine (Dacogen) and quisinostat (JNJ-26481585) were kindly provided by Johnson & Johnson (Beerse, Belgium) and used as a filter sterilized 10% hydroxypropyl-cyclodextran suspension.

### Treatment and isolation of murine 5T33MM cells

C57BL/KaLwRij mice were purchased from Harlan CPB (Horst, The Netherlands). Mice were housed according to the conditions approved by the Ethical Committee for Animal Experiments of the Vrije Universiteit Brussel (license no. LA1230281). The 5T33MM model was maintained as previously described [86]. At day 0, naive C57BL/KaLwRij mice were injected with 5×10^5^ 5T33MM cells. At established disease (day 16), mice were treated with decitabine (0.2mg/kg) (intraperitoneal injection, daily) or quisinostat (1.5mg/kg) (subcutaneous injection, once every other day). After 5 days, mice were sacrified. Bone marrow was isolated from hind legs and subjected to red blood cell lysis. For mRNA analysis, tumor cells were purified by depletion of CD11b+ contaminating cells. An overall mean purity of plasma cells of above 90% was obtained (Supplementary Figure S1C). Cytospins were made before and after depletion to count the percentage of plasma cells as described previously [32].

### Gene expression profiling

Samples with more than 95% plasma cells (N=4 in each group) were used for RNA isolation using the RNeasy Kit (Qiagen, Venlo, The Netherlands). RNA was further processed and hybridized to the Mouse Genome 430 2.0 Array (Affymetrix, Santa Clara, CA, USA) as described in Moreaux et. al [35]. Microarray data are available at ArrayExpress database (Accession number: E-MTAB-3178).

### Microarray data of primary multiple myeloma cells and human myeloma cell lines

Affymetrix data of two independent cohorts of previously untreated MM patients was used. The training cohort consists of 206 MM patients and is termed the Heidelberg-Montpellier (HM)-cohort. This cohort also includes 7 bone marrow plasma cell (BMPC) samples from healthy donors. The samples were obtained after written informed consent in accordance with the Declaration of Helsinki and after approval of the ethics committee of Montpellier and Heidelberg. These data are publically available through ArrayExpress database (E-MTAB-372). The validation cohort contains 345 MM patients from the University of Arkansas for Medical Sciences (UAMS, Little Rock, AR, USA) and is termed the TT2-cohort. These data can be accessed at the online Gene Expression Omnibus (GSE2658). In the HM study, patients underwent front line high-dose conditioning chemotherapy with 200mg/m² melphalan and autologous stem cell transplantation [5, 56]. In the UAMS study, patients received thalidomide or placebo in combination with 4 consecutive induction cycles containing (i) vincristine + doxorubicin + dexamethasone, (ii) cyclophosphamide + etoposide + cisplatin + dexamethasone, (iii) cyclophosphamide + doxorubicin, and (iv) cyclophosphamide + etoposide + cisplatin + dexamethasone (corresponding to total therapy 2 (TT2)). This was followed by high-dose melphalan and autologous stem cell transplantation [54, 87, 88]. The presence of cytogenetic abnormalities was evaluated by iFISH in the patients of the HM cohort. For TT2, the presence of t(4;14) was predicted by *MMSET* spike expression [89]. We also used Affymetrix data of 152 relapsed MM patients subsequently treated with bortezomib (GSE9782) from the study by Mulligan *et* al. [57]. The clinical characteristics of the cohorts have been described previously and are summarized in Supplementary Table S8 [54-57]. In addition, we used the Affymetrix data of 40 human myeloma cell lines (HMCLs) stored in the ArrayExpress database (E-TABM-937 and E-TABM-1088) [58]. The derivation of the HMCLs as well as their phenotypic and molecular characteristics were previously published [58].

### Statistical analysis and bio-informatics

Normalization of gene expression data was done by the MAS5 algorithm (scale 100) and analyzed by Significance of Microarray Analysis (SAM) [90] and bio-informatics platforms RAGE and Amazonia [91, 92]. The Benjamini-Hochberg multiple testing corrections were done for estimation of false discovery rate (FDR). The prognostic value of the genes in terms of overall survival (OS) was determined using the Maxstat R package. Significance was determined by log-rank test followed by Benjamin-Hochberg multiple testing correction. To combine the prognostic value of all those genes, a score was constructed and termed the murine DNA methylation (Mu-DM) and histone acetylation (Mu-HA) score. The scores are the sum of the Cox β-coefficients of the human orthologs of the decitabine- or quisinostat-deregulated genes with prognostic value in the training cohort, weighted by + or – 1 if the patients' MM cell MAS5 signal for any given gene is above or below the probe set Maxstat cutoff of this gene [35, 59, 89]. Results were plotted using the Kaplan-Meier method. Multivariate analysis of the scores was done using Cox proportional hazard model. The above analyses were performed using R 2.15.1 and Bioconductor 2.0 software. Gene ontology (GO) analysis was done by the online Database for Annotation, Visualization and Integrated Discovery (DAVID) [63, 64]. Pathway analysis was conducted by Reactome software, Pathway-Guide and STRING analysis.

## SUPPLEMENTARY MATERIAL FIGURES


















