# Supplementary material for: In vivo treatment with epigenetic modulating agents induces transcriptional alterations associated with prognosis and immunomodulation in multiple myeloma
Source: Oncotarget. 2014 Dec 26;6(5):3319–34. doi: 10.18632/oncotarget.3207 (PMC4413656; doi:10.18632/oncotarget.3207)
Supplement: Supplementary file 1 [file oncotarget-06-3319-s001.pdf]

In vivo treatment with epigenetic modulating agents induces transcriptional alterations associated with prognosis and immunomodulation in multiple myeloma

Supplementary Material

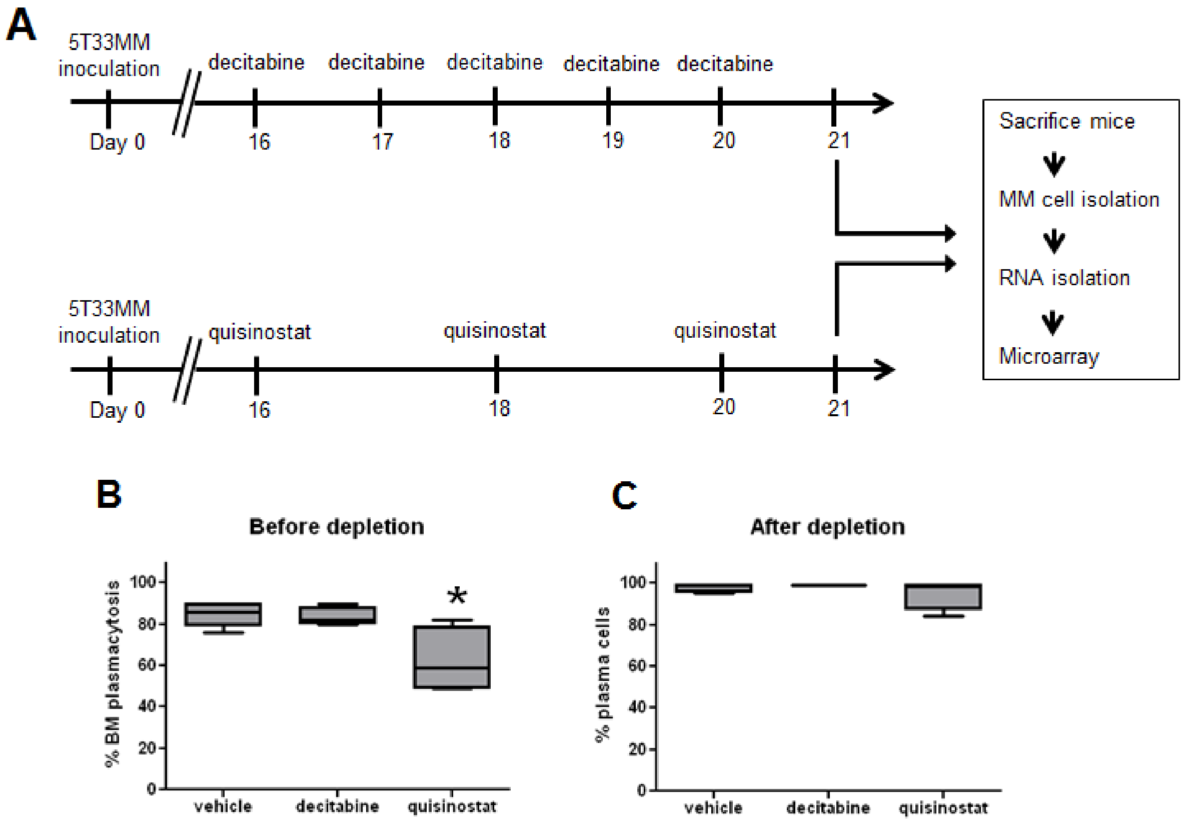

Supplementary Figure S1: A: Experimental treatment schedule. C57BL/KaLwRij mice were inoculated with 5T33MM cells and treated for 5 days starting at day 16. Treatment groups were vehicle (n=4), 0.2mpk decitabine (n=4) or 1.5mpk quisinostat (n=4). After sacrification, BM from hind legs was isolated. For mRNA analysis, tumor cells were purified by depletion of CD11b+ contaminating cells. RNA was isolated and processed for microarray analysis as described in Material and Methods. B, C: Cytospins were made and stained with May Grünwald-Giemsa and BM plasmacytosis was quantified by manual counting. B: % bone marrow plasmacytosis before CD11b depletion. * indicates $p<0.05$ compared to vehicle. C: % of plasma cells after CD11b depletion.

Term	p-value	n°
cell activation	9.710e-7	41
regulation of cell death	1.150e-6	66
regulation of programmed cell death	2.030e-6	64
regulation of locomotion	3.980e-6	38
regulation of cellular component movement	2.360e-5	37
regulation of apoptotic process	2.650e-5	61
regulation of cell motility	6.580e-5	34
mRNA processing	1.530e-4	30
programmed cell death	1.959e-4	54
regulation of cell migration	2.110e-4	32
apoptotic process	3.750e-4	53
cell death	3.750e-4	60
death	4.229e-4	60
response to alcohol	7.759e-4	23
response to wounding	1.190e-3	50
regulation of response to stimulus	2.530e-3	98
response to organic substance	2.579e-3	81
T cell differentiation	2.909e-3	14
response to cytokine	3.040e-3	34
response to chemical	3.109e-3	104
cellular response to chemical stimulus	3.200e-3	76
RNA splicing	3.490e-3	24
response to hydrogen peroxide	4.570e-3	13
cellular response to organic substance	5.649e-3	64
positive regulation of cell migration	7.369e-3	21
regulation of molecular function	7.860e-3	81
platelet activation	8.109e-3	19
mRNA metabolic process	1.250e-2	34
negative regulation of programmed cell death	1.250e-2	38
positive regulation of cellular component movement	1.319e-2	21
negative regulation of cell death	1.370e-2	39
regulation of catalytic activity	1.799e-2	69
response to stress	2.340e-2	99
positive regulation of locomotion	2.589e-2	21
cell surface receptor signaling pathway	2.680e-2	81
leukocyte activation	3.259e-2	24
T cell activation	3.370e-2	17
immune system process	3.429e-2	71
RNA splicing, via transesterification reactions	4.189e-2	17
lymphocyte differentiation	4.229e-2	16
regulation of cell proliferation	4.710e-2	54

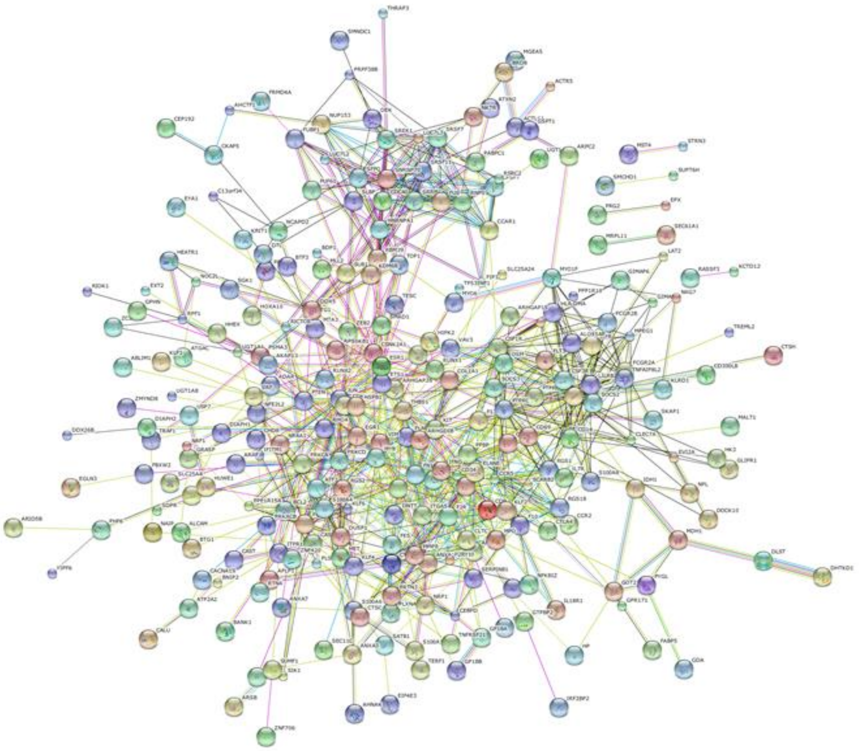

Supplementary Figure S2: STRING protein network analysis of quisinostat-deregulated genes. This scheme represents the gene product interactions. On the left, the main processes are summarized.

Term	p-value	n*
regulation of cell death	2.009e-6	29
regulation of apoptotic process	7.829e-6	27
regulation of programmed cell death	7.829e-6	27
cellular response to cytokine stimulus	3.010e-3	14
response to cytokine	3.650e-3	15
response to inorganic substance	3.650e-3	12
regulation of gene expression	7.429e-3	39
response to alcohol	8.999e-3	10
cytokine-mediated signaling pathway	1.099e-2	11
positive regulation of apoptotic signaling pathway	1.389e-2	7
RNA splicing	2.560e-2	10
response to stress	2.560e-2	34
response to hydrogen peroxide	2.659e-2	6
mRNA processing	2.659e-2	11
regulation of apoptotic signaling pathway	2.659e-2	10
regulation of macromolecule metabolic process	2.659e-2	42
regulation of growth	2.659e-2	13
cellular response to cycloheximide	2.659e-2	2
neutrophil aggregation	2.659e-2	2
defense response	2.790e-2	20
response to organic substance	3.070e-2	27
response to ethanol	4.000e-2	6
regulation of primary metabolic process	4.710e-2	43
response to lipid	4.710e-2	13
response to molecule of bacterial origin	4.710e-2	8
response to zinc ion	4.710e-2	4
response to chemical	4.710e-2	33

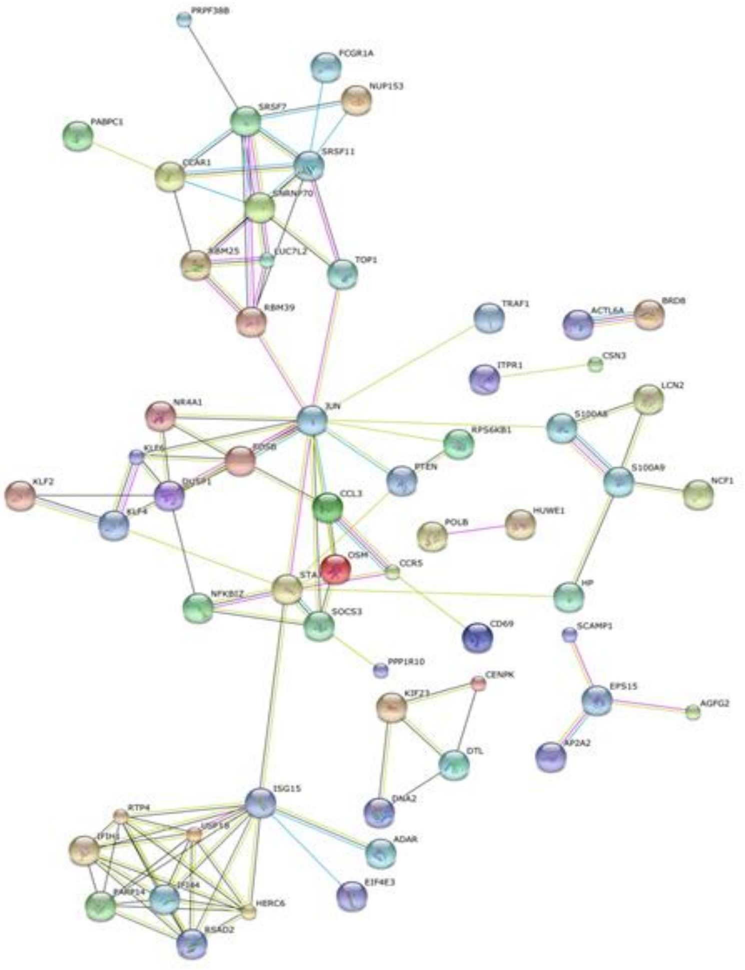

Supplementary Figure S3: STRING protein network analysis of decitabine-deregulated genes. This scheme represents the gene product interactions. On the left, the main processes are summarized.

Supplementary Tables

Supplementary Table S1: List of the decitabine-deregulated genes (Benjamini-Hochberg < 0.05, ratio >2).

Supplementary Table S2: List of the quisinostat-deregulated genes (Benjamini-Hochberg < 0.05, ratio >2).

Supplementary Table S3: Predictive power of the Mu-HA score to predict response to bortezomib of relapsed patients. R=responders (CR + PR + MR). NR = non-responders (NC + PD). PPV = positive predictive value. NPV = negative predictive value. P value was calculated by the Fisher exact test.

Supplementary Table S4: Cox univariate and multivariate analysis of OS in HM and TT2 patients' cohorts. The prognostic factors were tested as single variable or multi variables using Cox-model. P-values and the hazard ratios (HR) are shown. NS, Not significant at a 5% threshold; GPI, gene expression based proliferation index; ISS, International Staging System; HRS, high-risk score; IFM, Intergroupe Francophone du Myélome; RS, Risk Score. Translocation t(4;14) was predicted in the TT2-corhort as described in material and methods. NA, not available.

Supplementary Table S5: List of gene ontology terms according to DAVID software (Ontology database: GOTERM_BP_FAT) associated with deregulated genes after treatment with quisinostat or decitabine.

Supplementary Table S6: List of pathways according to Reactome pathway analysis associated with the deregulated genes after quisinostat or decitabine treatment.

Supplementary table S7: Top list of enriched processes and pathways depicted by Pathway-Guide after treatment with quisinostat or decitabine.

Supplementary table S8: Clinical patient data for age, beta-2-microglobulin, and bone marrow plasma cell infiltration in the HM-, TT2 and Mulligan-cohort. Median value and range are given. NA, not available. ISS, International Staging System.
